# T152. NRN1 GENE AND FUNCTIONAL MRI: ASSOCIATION ANALYSIS IN SCHIZOPHRENIA PATIENTS AND HEALTHY SUBJECTS

**DOI:** 10.1093/schbul/sby016.428

**Published:** 2018-04-01

**Authors:** Carmen Almodóvar, Maria Guardiola, Pilar Salgado-Pineda, Marcos Moreno, Carme Gallego, Claudia Prats, Barbara Arias, Edith Pomarol-Clotet, Peter J McKenna, Mar Fatjó-Vilas

**Affiliations:** 1 FIDMAG Research Foundation, University of Barcelona; 2 FIDMAG Research Foundation. CIBERSAM, University of Barcelona; 3 FIDMAG Research Foundation. CIBERSAM; 4 Molecular Biology Institute of Barcelona (IBMB-CSIC); 5 University of Barcelona, CIBERSAM

## Abstract

**Background:**

Alterations of synaptic plasticity are currently accepted to play a critical role in schizophrenia (SZ). Among genes of neuronal plasticity there is Neuritin1 gene (NRN1), which has been associated with SZ, age at onset and differences in general cognitive performance in this disorder. However, little is known about the brain imaging correlates of NRN1 gene. We aimed: i) to investigate the association of NRN1 with schizophrenia-spectrum disorders (SZ-SD), exploring its role in age at onset through a family-based study, ii) to examine the brain functional correlates of NRN1 sequence variants through a neuroimaging genetics approach using a case-control design.

**Methods:**

A family-based association analysis was carried out with a sample of 588 individuals from 159 families (74 early onset / 85 adult onset) with an offspring with a diagnosis of SZ-SD. An independent sample consisting of 45 subjects (26 patients / 19 controls) was used to perform a case-control neuroimaging genetics analysis. DNA was extracted from blood/buccal mucosa samples and eleven Single Nucleotide Polymorphisms (SNPs) in NRN1 were genotyped. The linkage disequilibrium between the SNPs was estimated in the family-based sample with Haploview v4.1. Three haplotype blocks were defined: 1) rs2208870, rs12333117, rs582186, 2) rs645649, rs582262, 3) rs3763180, rs10484320, rs4960155, rs9379002, rs9405890, rs1475157. PLINK-v1.06 was used for the tabulation of possible individual haplotype phases and for the family-based association analyses (Transmission Disequilibrium Test). To explore the brain functional correlates of NRN1, the subjects belonging to case-control sample underwent a single MRI scanning session and performed a virtual reality spatial navigation task (Salgado-Pineda et al. 2016). The standard atlas provided in the FSL package was used to define three separate ROIs (left and right hippocampus and medial frontal region (mPFC)) and the mean value of activation per each subject was used to test the effect of each SNP/haplotypes by means of a linear regression. All the analyses were adjusted by age, sex and premorbid intelligence coefficient (IQ-TAP).

**Results:**

Two haplotypes including SNP4 and SNP5 (rs645649-rs582262) were associated with early onset SZ-SD: the haplotype CG was undertransmitted from parents to patients (p=0.011, OR (95%CI=0.08(0.01–0.71) - protective haplotype), while the haplotype GG showed an overtransmission trend (p=0.055, OR (95%CI=3.83 (1.40–10.48)). No effect was observed in the adult onset subsample.

No differences between patients and controls were observed in the activation of the three ROIs. Within patients, an effect of the haplotype CG (SNP4-5) was detected in the mPFC: carriers of no copies of the protective haplotype showed a higher mean activation (n=15, mean(SD)=-1.17(17.37)) than individuals with at least one copy of the haplotype (n=9, mean(SD)=-21.19(21.94)) (⎕=-0.507 p=0.035).

**Discussion:**

First, our family-based results are consistent with evidence of a genetic association between NRN1 gene and SZ-SD and extend the knowledge on that NRN1 has a selective impact on early age at onset (Fatjó-Vilas et al. 2016). Second, our data suggest that NRN1 is involved in the regulation of the de-activation of mPFC in patients with SZ during a spatial navigation task. This result is of special interest since mPFC is an area included in the Default Mode Network (DMN) and alterations in this network have been highly documented in SZ patients during performance of different tasks (Pomarol-Clotet et al. 2008; Mannell et al. 2010; Salgado-Pineda et al. 2011).

**Acknowledgements:**

Fundación Alicia Koplowitz.

